# Impact of Highland Topography Changes on Exposure to Malaria Vectors and Immunity in Western Kenya

**DOI:** 10.3389/fpubh.2016.00227

**Published:** 2016-10-14

**Authors:** Christine Ludwin Wanjala, Eliningaya J. Kweka

**Affiliations:** ^1^Centre for Global Health Research, Kenya Medical Research Institute, Kisumu, Kenya; ^2^Department of Medical Laboratory Sciences, Masinde Muliro University of Science and Technology, Kakamega, Kenya; ^3^Mosquito Section, Division of Livestock and Human Diseases Vector Control, Tropical Pesticides Research Institute, Arusha, Tanzania; ^4^Department of Medical Parasitology and Entomology, Catholic University of Health and Allied Sciences, Mwanza, Tanzania

**Keywords:** topography, V-valley, U-valley, immunity, exposure, rapid diagnostic kit, highland

## Abstract

**Background:**

It is almost an axiom that in the African highlands (above 1,500 m) transmission of *Plasmodium falciparum* is limited primarily by low ambient temperature and that small changes in temperature could result in temporary favorable conditions for unstable transmission within populations that have acquired little functional immunity. The pattern of malaria transmission in the highland plateau ecosystems is less distinct due to the flat topography and diffuse hydrology resulting from numerous streams. The non-homogeneous distribution of larval breeding habitats in east African highlands obviously affects *Anopheles* spatial distribution which, consequently, leads to heterogeneous human exposure to malaria. Another delicate parameter in the fragile transmission risk of malaria in the highlands is the rapid loss of primary forest due to subsistence agriculture. The implication of this change in land cover on malaria transmission is that deforestation can lead to changes in microclimate of both adult and larval habitats hence increase larvae survival, population density, and gametocytes development in adult mosquitoes. Deforestation has been documented to enhancing vectorial capacity of *Anopheles gambiae* by nearly 100% compared to forested areas.

**Method:**

The study was conducted in five different ecosystems in the western Kenya highlands, two U-shaped valleys (Iguhu, Emutete), two V-shaped valleys (Marani, Fort Ternan), and one plateau (Shikondi) for 16 months among 6- to 15-year-old children. Exposure to malaria was tested using circumsporozoite protein (CSP) and merozoite surface protein immunochromatographic antibody tests. Malaria parasite was examined using different tools, which include microscopy based on blood smears, rapid diagnostic test based on HRP 2 proteins, and serology based on human immune response to parasite and vector antigens have been also examined in the highlands in comparison with different topographical systems of western Kenya.

**Results:**

The results suggested that changes in the topography had implication on transmission in highlands of western Kenya and appropriate diagnosis, treatment, and control tool needed to be considered accordingly. Both plateau and U-shaped valley found to have higher parasite density than V-shaped valley. People in V-valley were less immune than in plateau and U-valley residents.

**Conclusion:**

Topography diversity in western Kenya highlands has a significant impact on exposure rates of human to malaria vectors and parasite. The residents of V-shaped valleys are at risk of having explosive malaria outbreaks during hyper-transmission periods due to low exposure to malaria parasite; hence, they have low immune response to malaria, while the U-shaped valleys have stable malaria transmission, therefore, the human population has developed immunity to malaria due to continuous exposure to malaria.

## Introduction

Malaria is a major human health threat that occurs globally in tropical and sub-tropical regions. Though malaria has been declining from 1.5 million mortality cases to 438,000 cases in 2014 (1), efforts has to be made to investigate the underlying causes of these hundreds of death cases. The mounting threat is evidence witnessed by increased numbers of malaria outbreaks in the Kenyan highlands (elevation >1,500 m), where malaria was previously rare (2, 3). Malaria outbreaks occurred sporadically in the highlands of western Kenya from the 1920s to the 1950s (4, 5). Since 1988, however, malaria epidemics have occurred with heightened frequency throughout the Kenyan highlands, causing serious mortality and morbidity (2, 3). Malaria in the Western Kenya Highland is characterized by unstable and high-transmission variability (6, 7), which results into epidemics during periods of suitable climatic conditions. Depending on drainage characteristics of the highland vector ecosystems, different transmission intensities results into heterogeneous exposure and development of immunity (6, 8–10). The sensitivity of a site to malaria epidemics is dependent on the level of immunity of the human population. In the last two decades, malaria epidemics have increased in frequency and intensity in the East Africa highlands in populations that have little or no exposure to plasmodial infections (3, 11–13). Research indicates that the mechanisms leading to epidemic malaria in the highlands are complex and are probably due to the concerted effects of factors, such as topography, hydrology, climate variability, land-use/land-cover change, and drug resistance (6, 8–10, 14). Effective disease control calls for a clear understanding of the interaction between these epidemiologic factors (9).

In this study, the highlands topography was classified into three ecosystems these being the flat bottomed valleys (U-shaped), the narrow bottomed valley (V shaped), and the plateau. A longitudinal cohort study was carried out with a primary focus on a spatial-temporal qualitative assessment of exposure to infections using immunological markers in the different ecosystems. Parasitological surveys were carried out to provide baseline data on the effects of ecosystem characteristics on malaria prevalence. Malaria epidemics in western Kenya highlands are driven by climate variability. However, environmental terrain characteristics can modify the level of malaria transmission and the rate of development of immunity (10). Immunity to severe malaria generally requires only a few infections at any level of endemicity (15–17). However, the long interval between infections and the spatial variability of transmission in areas of unstable endemicity fail to provide frequent enough challenge to sustain much disease-modulating immunity. Recent studies suggested that residents of highland areas generally lack immunity to *Plasmodium falciparum* and are particularly vulnerable to malaria infection (18–20). The proportion of asymptomatic individuals is usually lower in highlands than in high-transmission areas where there is small among-season variation in *P. falciparum* prevalence and parasite densities (21); thus, a small increase in the abundance of vectors may lead to a significant malaria outbreak in the highlands. At high altitudes in the highlands and on hilltops, where malaria transmission intensity is low, human populations have poorly developed immunity to malaria because exposures are infrequent, and persons are vulnerable to severe clinical illness and complications from *Plasmodium* infection (22). High risk for severe malaria is seen in persons living in areas with low-to-moderate transmission intensities (23). In such areas, the proportion of asymptomatic persons is usually lower than in high-transmission areas, where *P. falciparum* prevalence and parasite density varies little between seasons (24). As a consequence of low immunity in the human population of the highlands, malaria epidemics have caused significant human mortality (6). Compared to the malaria situation between the 1920s and 1950s, the current pattern of malaria epidemics in the highlands is characterized by increased frequencies (23), expanded geographic areas (8, 25), and increased case-fatality rates (3). The reemergence of epidemic malaria is likely due to local malaria transmission in the highlands (2, 7, 26, 27). In the late 1980s and early 1990s, a series of malaria epidemics were reported in the western highlands of Kenya and other communities at high altitude in Africa (28–30). Whereas substantial progress has been made on epidemiology and ecology of malaria in highlands, little is known on what proportion of human population exposed to malaria has mounted an immune response. This study was designed to identify how major environmental terrain characteristics that control the breeding of malaria vectors in the western Kenya highlands can influence exposure to transmission and the development of an immune response.

## Materials and Methods

### Study Sites

Study sites where characterized into three topographical types.

#### “U”-Shaped Valleys

##### Iguhu in Kakamega

**Figure 1:**
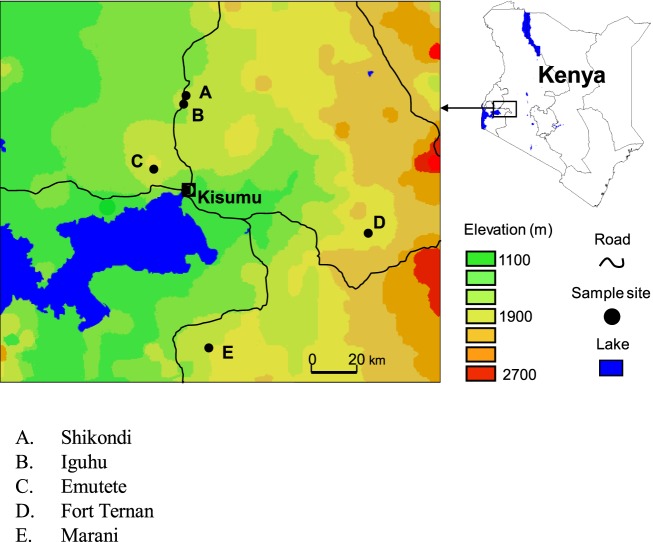
**A map showing the location of the study sites**.

Iguhu village (00°17′ N, 34°74′ E and elevation 1,450–1,580 m above sea level) is located in Kakamega district, western Kenya, with population ≈11,000 (Figure 1). This area experiences two rainy seasons and averages 2,000 mm rainfall per year. The long rainy season usually occurs between April and May, with an average monthly rainfall 150–260 mm, while the short rainy season usually occurs between September and October, with an average monthly rainfall 165 mm. Malaria prevalence peaks usually lag 1–2 months after the rain. The mean annual daily temperature is 20.8°C. The area has experienced extensive deforestation and swamp reclamation in recent years as a result of rapid human population growth and the demand for settlement and agricultural land; therefore, only patches of forest remain. Malaria vectors in the area are *Anopheles gambiae* sensu stricto and *Anopheles funestus* (31–33). Maize is the principal subsistence crop with vegetables grown on small irrigated plots in valley bottoms. The Yala River bisects the area; most mosquito larval habitats are found on riverbanks in the bottom of the valley and on the banks of streams during both dry and rainy seasons (34).

##### Emutete in Emuhaya

Emutete village (00°22′ N, 34°64′ E and elevation 1,463–1,603 m above the sea level) is located in Emuhaya district (Figure 1). This area is hilly characterized by steep-sided valleys with flat bottoms and plateaus where most homes are built. Small streams run along the valley bottoms and papyrus swamps are common. The valleys are approximately 2.4 km^2^ in area and at least 1 km apart. This area is densely populated, with malaria prevalence of 39.6% (35). This area experiences two rainy seasons and averages 2,500 mm rainfall per year. Malaria prevalence peaks usually lag 1–2 months after the rain. The mean annual daily temperature is 20.5°C.

#### “V”-Shaped Valleys

##### Marani in Kisii

Marani (00°02′ N, 34°48′ E and elevation 1,520–1,700 m above the sea level) is located on the highland plateau adjacent to the Lake Victoria Basin and 17 km north of Kisii (Figure 1). This area is in a steep valley where the Marani River flows through a shallow gorge on the western side, and river banks are forested with *Eucalyptus* trees. Malaria epidemics have occurred frequently in this area in the last 15 years.

##### Fort Tenan in Kericho

Fort Ternan (00°12′ S, 35°21′ E and elevation 1,500–1,600 m above the sea level) is found in Kericho District (Figure 1). Malaria prevalence in this area is unstable varying annually between 10 and 60%, due to this instability, epidemics are common with a high morbidity and mortality rates. Depending on the temperature during a specific year, transmission risk in this area is either seasonal or sporadic (2). This area consists of a variety of land covers ranging from shore regions to hilly areas and from rainy forests to tea plantations. Agricultural activities in these area are dominated by cash crops, e.g., sugarcane, tea, and coffee.

#### Plateau

##### Shikondi in Kakamega

Shikondi (00°19′ N, 34°76′ E, elevation 1,533–1,576 m above the sea level) is a plateau found in Kakamega district, Western Kenya (Figure 1). This area experiences two rainy seasons and averages 2,000 mm rainfall per year. The long rainy season usually occurs between April and May, with an average monthly rainfall 150–260 mm, while the short rainy season usually occurs between September and October, with an average monthly rainfall 165 mm. Malaria prevalence peaks usually lag 1–2 months after the rain. The mean annual daily temperature is 20.8°C.

### Laboratory Analysis

#### Immunoassay for Exposure to Malaria

Exposure to malaria parasites is indicated by the presence of circumsporozoite proteins (CSPs) antibodies and the merozoite surface protein (MSP) antibodies in the serum. These antibodies persist even if the individual is cured of the parasite. Malaria P.f./P.v. 3 line test device consist of a sample window containing an absorbent pad where the serum/plasma are to be added. It contains a membrane strip, which is pre-coated with recombinant malaria P.f. capture antigens (MSP, CSP) on the test band P.f. region and with recombinant malaria P.v. capture antigens (MSP, CSP) on the test band P.v. region. The recombinant malaria P.v./P.f. antigen (MSP, CSP)-colloidal gold conjugate and serum sample moves along the membrane chromatographically to the test region (P.f., P.v.) and forms a visible line as antibody–antigen gold particle complex forms with high degree of sensitivity and specificity. Blood samples were collected by a standard finger prick and put in capillary tubes, then centrifuged at 1,800 rpm for 5 min to obtain the serum. After the test, kit had been brought to room temperature, the device was put on a flat surface and a drop of serum was added in to the sample window and allowed to soak in, then two drops of the diluent was added into the sample window. The positive results were read in 10 min and the negative results in 20 min.

#### Microscopic Analysis of Malaria Parasites

Standard finger-prick method was used for blood samples collection and thick and thin smears prepared on well labeled slides. After smears were air dried, they were fixed in methanol and stained with 4% Giemsa for 30 min (36). The stained smears were then examined using the magnification of 1,000× oil immersions to identify and count the parasite species. Random checks were carried out on the slide counts by independent microscopists to ensure quality control. Parasite density was scored against leukocytes (8,000 cells/μl of blood) positive slide; otherwise, the whole slide was carefully scanned before being declared negative (37).

### Weather Data

Meteorological data collected were mean monthly rainfall, maximum, and minimum temperature for each study site. Data were obtained from the Kenya Department of Meteorology.

### Data Analysis

Malaria parasites site-specific prevalence rates were determined by expressing positive blood smears as a percentage of all the examined blood smears. Geometric mean parasite density was calculated for each site considering only true positive slides. The paired *t*-test was used to determine differences of the prevalence of antibody and malaria parasites infections among the sites at 0.05 significance level. *K* function was used to determine if spatial distribution of infections in the sites was significantly clustered or it was random. In this method, the distance interval is specified, and then the GIS calculates the average number of infections within the distance of each infection. Increasing distances at a specified interval shows at what distance the concentration of infections is the greatest. If the average number of infections found at a distance is greater than that for a random distribution throughout the study area, then the distribution is considered clustered. The GIS finds the distance from each point to every other point, and then, for each point, counts up the number of surrounding points within the given distance. *I* is a weight and is either 1 if the neighboring point is within the distance of the target point or 0 if it is not. To see if there is a pattern, the observed *K* values at each distance is compared to the expected values for a random distribution. This is done by plotting the values on the chart, with the K values on the *y*-axis against the distance on the *x*-axis. The *K* value gets very large as the distance increases to reduce the height of the *y*-axis and make the chart easier to read, log transformed values are plotted instead of the raw *K* values. *L* (*d*) is a variation of the *K* values. At any distance, if the line for the observed *L* values is above that of the expected values, the distribution is more clustered than expected for random distribution. If it is below the line for the expected values, the distribution is more dispersed. To determine if the pattern is statistically significant, the curve of the observed distribution is compared to the confidence limits for a random distribution. An observed *L* value that exceeds the upper confidence limit indicated statistically significant clustered pattern for that distance, while one that falls below the lower limit indicates a statistically significant dispersed pattern (38).

## Results

### Malaria Parasite Prevalence

Both *P*. *falciparum* and *Plasmodium malariae* was found in Iguhu and Emutete while only *P. falciparum* was found in the other sites. Malaria parasites prevalence varied significantly between the U-shaped and the V-shaped valleys (*t* = 12.036, df = 12, *P* < 0.05), between U-shaped valley and the plateau (*t* = −11.65, df = 12, *P* < 0.05) and between V-shaped valleys and the plateau (*t* = −2.76, df = 12, *P* < 0.05). There was no significant difference in the parasite prevalence within the U-shaped valleys (*t* = 1.27, df = 12, *P* > 0.05), and within the V-shaped valleys (*t* = −4.44, df = 12, *P* > 0.05). The mean parasite prevalence in the U-shaped valleys was 22.12%, in the V-shaped valleys was 2.76% and at the plateau was 4.42% over the study period. There was 5.0-fold greater parasite prevalence in the U-shaped compared to the V-shaped valleys.

### Parasite Density

Population living in U-shaped valleys had higher parasite density compared to the V-shaped valleys but varied at the plateau. Geometric means varied significantly between the V-shaped valleys and the plateau (*t* = −2.72, df = 12, *P* < 0.05) and between the V-shaped valleys and the U-shaped valleys (*t* = −7.03, df = 12, *P* < 0.05). There was no significant difference in the geometric means within the V-shaped valleys (*t* = −2.1, df = 12, *P* > 0.05), within the U-shaped valleys (*t* = −1.73, df = 12, *P* > 0.05) and between the U-shaped valley and the plateau (*t* = 1.38, df = 12, *P* > 0.05).

### Gametocyte Prevalence

**Figure 2:**
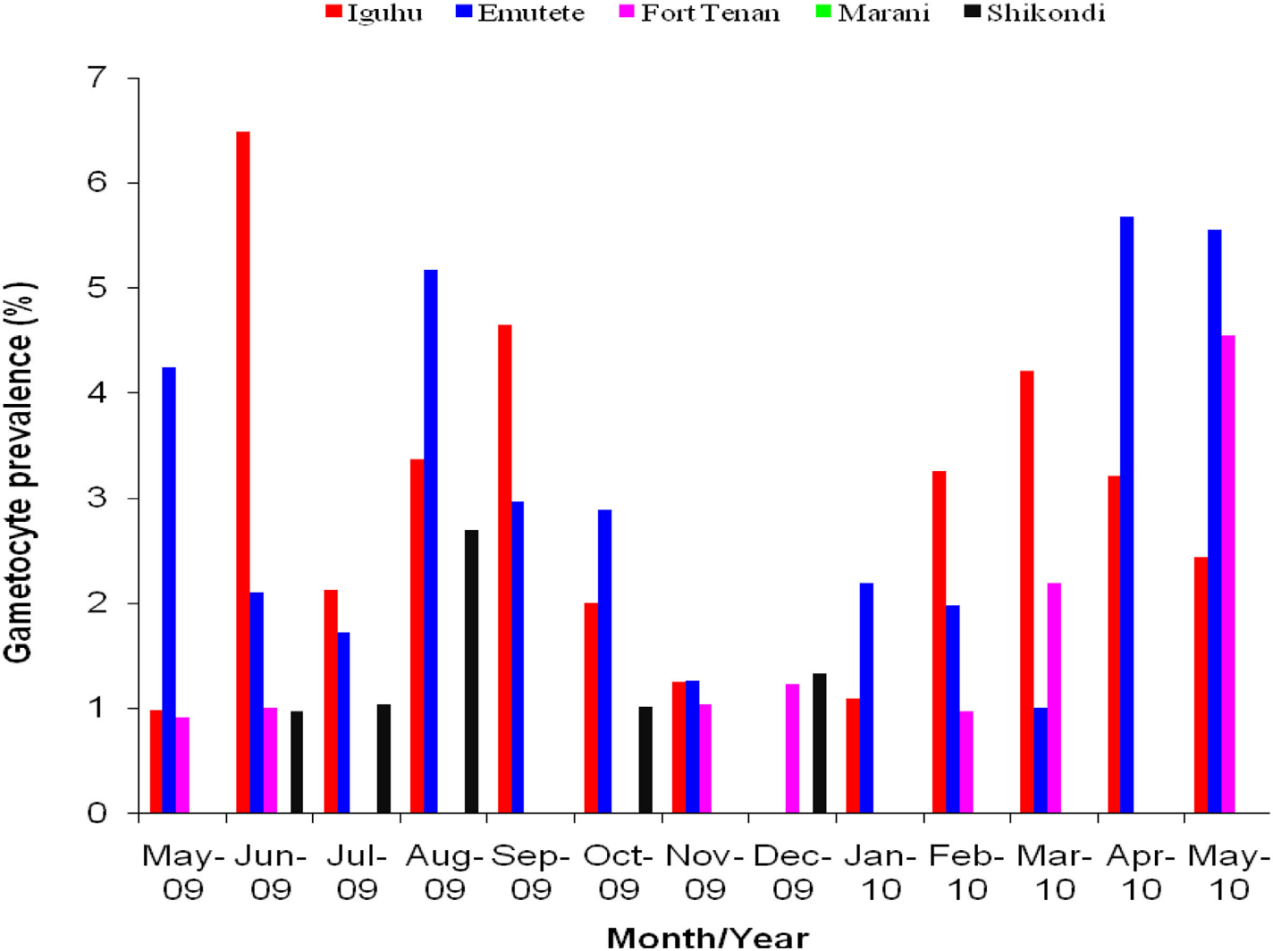
**Gametocytes prevalence rates in the five sites, no gametocytes were observed at Marani, Kisii during the period of study**.

The U-shaped valleys had higher *P. falciparum* gametocytes prevalence compared to the V-shaped valleys and at the plateau. Gametocytes prevalence varied significantly between the V-shaped valleys and the U-shaped valleys (*t* = 6.60, df = 12, *P* < 0.05), between V-shaped valleys and the plateau (*t* = 3.13, df = 12, *P* < 0.05) and between U-shaped valleys and the plateau (*t* = 3.34, df = 12, *P* < 0.05). There was no significant difference in the gametocytes prevalence within the V-shaped valleys (*t* = 0.30, df = 12, *P* > 0.05), within the U-shaped valleys (*t* = −0.20, df = 12, *P* > 0.05). No gametocytes found in Marani during the period of the study (Figure 2).

### Antibodies Prevalence

The U-shaped and V-shaped valleys had significant variations on antibodies prevalence varied between them (*t* = 6.23, df = 12, *P* < 0.05) and between the U-shaped valleys and the plateau (*t* = 6.18, df = 12, *P* < 0.05). There was no significant difference in the antibodies prevalence within U-shaped valleys (*t* = −0.66, df = 12, *P* > 0.05), within V-shaped valleys (*t* = 0.68, df = 12, *P* > 0.05) and between V-shaped valleys and the plateau (*t* = −0.99, df = 12, *P* > 0.05). The mean antibody prevalence in the U-shaped valley was 24.19%, in the V-shaped valley was 10.13% and at the plateau was 12.23% over the period of the study. There was 2.0-fold greater antibody prevalence in the U-shaped compared to the V-shaped valleys.

### Spatial Distribution of Malaria Infections

**Figure 3:**
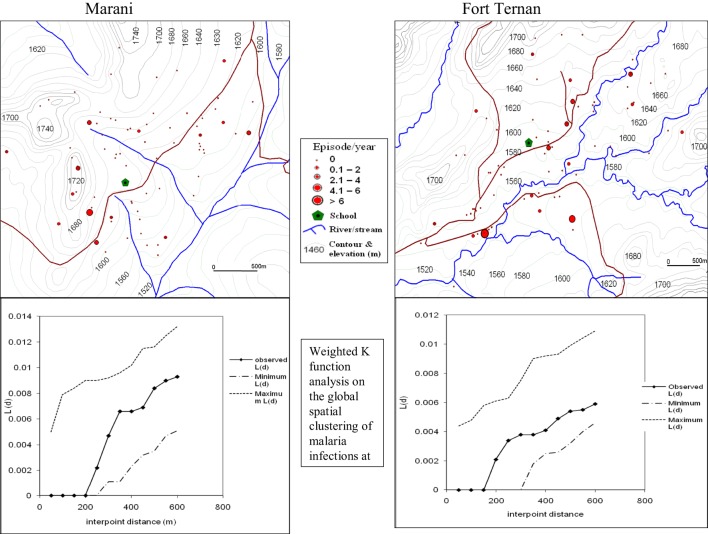
**Showing spatial distribution of malaria infections and cluster analysis in the “V”-shaped valleys of Marani and Fort Ternan (Weighted *K* function analysis shows that there was no significant clustering of malaria infections in Marani and Fort Ternan)**.

**Figure 4:**
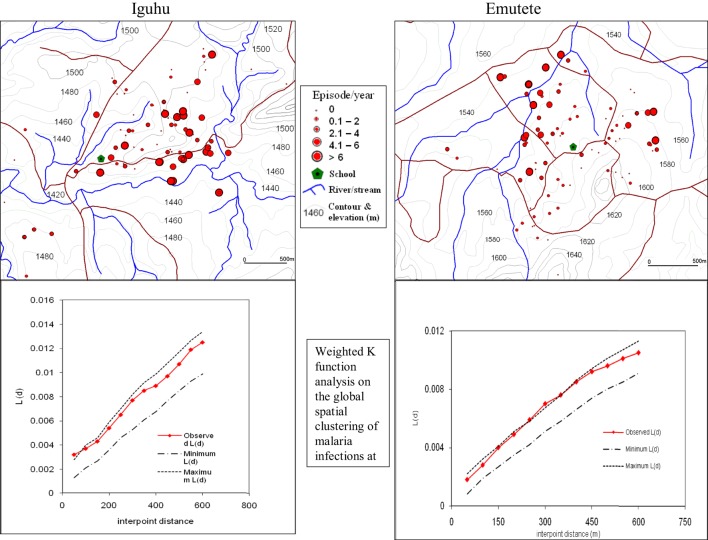
**Showing spatial distribution of malaria cases and cluster analysis in the “U” shaped valleys of Iguhu and Emutete**. The infections were clustered around low altitude areas and also around swamps (Weighted *K* function shows significant clustering of malaria infections in Emutete and Iguhu).

**Figure 5:**
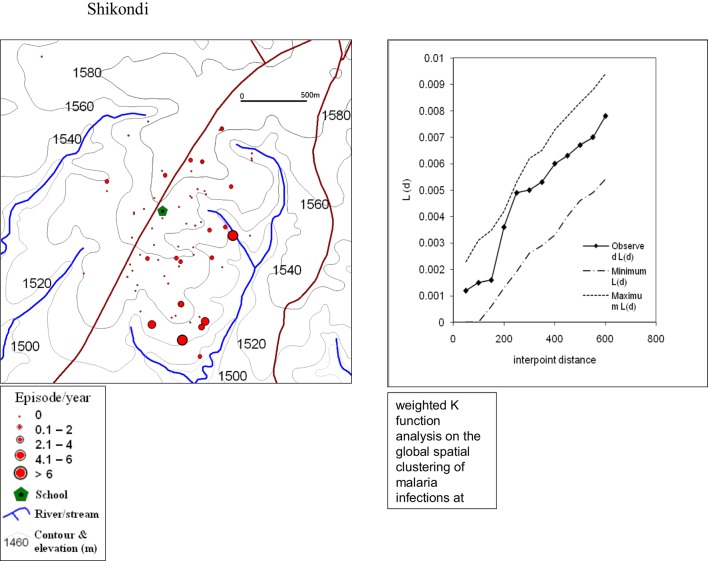
**Showing spatial distribution of malaria cases and cluster analysis from the Shikondi plateau**. The infections were randomly distributed with a few clusters around the low altitude (Weighted *K* function analysis indicates that there was no significant clustering of malaria infections at the plateau).

The global weighted *K* function, *L* (*d*), was used to examine the spatial distribution of malaria infection by household over an interpoint distance of 100–1,400 m for all the sites. Figure 3 shows measures of the observed *L* (*d*) and the 95% CI plotted for various values of interpoint distance for the Surveys in Emutete and Iguhu, respectively. The spatial distribution of infections is considered evenly dispersed if the observed K function values are below the lower limit of the 95% CI, clustered if above the upper limit, or random if within the 95% CI. The weighted *K* function indicated that the malaria infection distribution pattern was significantly different than expected under complete spatial randomness in the U-shaped valley (Figure 4) but was random in the V-shaped valley (Figure 3) and the plateau (Figure 5). Spatial clustering occurred at the swamps in the U-shaped valleys (Figure 4), it was less clustered at the plateau at low altitude but random in the V-shaped valley (Figure 3). The majority of children with parasite positive blood smears in the U-shaped valleys were found infected 4–6 occasions during 12 months, while those in the V-shaped valleys and the plateau had 1–2 infections per year. Thus, the children in the U-shaped valleys were infected for a longer period (Figures 3 and 4).

## Discussion

This study has revealed that the western Kenya highland topography is a complex ecosystem comprising of hills, plateaus, valleys, rivers, streams, and swamps. This ecosystem has profound effects on the level of malaria transmission. It has been shown that malaria transmission in the highlands is heterogeneous with the highest incidence being found at valley bottoms followed by hillsides and hilltops (9, 10, 14, 39). In this study, we found that the prevalence of malaria infections was fivefold higher in children living in the U-shaped valleys than in the children living in the V-shaped valleys and the prevalence of malaria infections at the plateau was similar to that of the V-shaped valleys. Similar findings have been reported in different topography in highlands (10, 16, 40, 41). The prevalence of malaria parasites antibodies was twofold higher in the children living in the U-shaped valleys than in the children living in the V-shaped valleys. Similar findings have been shown in western Kenya highlands with serological markers, where the residents at the valley bottom are more exposed to mosquitoes infectious bites than those living in uphill’s (42, 43). The gametocytes prevalence was also high in the children living in the U-shaped valleys compared to the V-shaped valleys and the plateau with no gametocytes observed at Kisii. The children population in U-valleys previously were found to have higher exposure to mosquitoes that subsequently have caused them to be in higher risk of *P. falciparum* gametocytes carriage (10, 16, 42, 43). Parasite densities were high in the children living in the U-shaped valley compared to the V-shaped valleys and varied greatly at the plateau. Similar findings were present previously with the human population in broad valleys having more exposure to mosquitoes and higher infection rates (9, 10, 44, 45). We also found out that there was clustering of infections at the swamps in the U-shaped valleys but the infections were randomly distributed in the V-shaped valley and the plateau. Topographic features of the highlands restrict the spatial distribution of vector breeding habitats; this, ultimately, affects malaria transmission, exposure of the human population to malaria infection, and development of immunity of human populations to malaria (9, 43, 45).

In the highlands, most severe malaria cases during an epidemic come from uphill human populations that have not been regularly exposed to malaria infection (7, 10, 44, 46–48). Studies of malaria in highland areas have generally concentrated on malaria parasites densities and vector densities in malaria stable areas within the highlands. Literature searches did not reveal studies that have assessed the immune profile of the population in the highlands and whether topography especially the shape of the valleys in the highlands and plateaus are consistently associated with malaria risk. Our study addresses this gap in knowledge and demonstrates that topography in this case the shape of the valley and plateaus affects exposure of the human population to malaria and immune response to malaria. These findings suggest that within the highlands some areas have stable malaria transmission and the human population in these areas has developed immunity to malaria; in low and unstable malaria transmission areas, the human population lack immunity to malaria and are likely to suffer severe clinical malaria and subsequently malaria epidemics.

High malaria parasites prevalence in the U-shaped valleys compared to the V-shaped valleys indicates that malaria transmission in the U-shaped valley is more stable. Our findings are similar to later studies which indicated that children in the malaria stable transmission areas have asymptomatic parasitemia and that children under this condition are able to suppress high parasitemia and presumably avoid severe disease (9, 45). The twofold higher mean antibodies prevalence than the mean parasites prevalence in the V-shaped valleys may suggest that malaria in the V-shaped valley is symptomatic, most cases are treated and more antibodies are detected since the parasites are cleared by the drugs. Other studies have shown a log-linear relationship between exposure and child prevalence predicted by mathematical models assuming exposure-dependent immunity (49). The log-linear relationship between transmission intensity and prevalence of both infections and enlarged spleens, thus, support the existence of exposure-dependent acquired immunity (50). Our results also indicate that children in the U-shaped valley were able to maintain the same level of parasite densities throughout the study, but children in the V-shaped valley took long to achieve the same levels of parasite densities as those in the U-shaped valleys, this suggests that children in stable malaria transmission areas are able to control the numbers of parasites in the blood during a period of increased exposure to infected mosquitoes in the main transmission season (10, 42, 43). Apart from *P. falciparum, P. malariae* was observed in the U-shaped valleys but only *P. falciparum* was observed in the V-shaped valleys and the plateau, this suggest that residents of malaria stable transmission areas often have mixed infections but only *Plasmodium* species is found in unstable malaria transmission areas (51).

There was also great variation of the parasites densities at the plateau that is indicated by the magnitude of the error bars, this can be explained by the flat topography and the more diffuse hydrology resulting from numerous streams at the plateau. Other studies have shown that malaria transmission at the plateau ranges from low on the hilltops to high to a level as high as that at the valley bottoms (10, 42, 44). According to the Ministry of Health in Burundi, malaria in the high plateau is hypo- to meso-endemic and prone to epidemics (52). Our results have shown clearly that malaria prevalence in the highland is greatly affected by the terrain characteristics of the highland, the dynamics of malaria infections were significantly different among the two valley systems and the plateau indicating that malaria transmission in the highlands is heterogeneous due to its complex topographical features. The shape of the valley affects the availability of mosquito breeding habitats, thus affecting malaria transmission. U-shaped valleys have poor drainage and this causes accumulation of water during rainy seasons creating excellent breeding habitats for malaria vectors; by contrast, there is no accumulation of water in the V-shaped valleys due to good drainage.

The high gametocytes prevalence rates in the U-shaped valleys compared to the V-shaped valleys indicate that the reservoir of malaria infections in the U-shaped valleys is higher than in the V-shaped valleys and the plateau. While the population living in the U-shaped valley maintains a large reservoir of infectious gametocytes, the people living in the V-shaped valley comprise a high proportion of susceptible individuals. Under permissive climatic conditions, the infectious vector population could increase, leading to higher rates of malaria prevalence. In our study, the mean gametocyte density varied significantly between the V-shaped and the U-shaped valleys, and between each of the two valley systems and the plateau. The low prevalence of gametocytes in the V-shaped valleys with no gametocytes observed in Kisii indicates that there is a weak transmission system in the V-shaped valley (15, 16).

High antibody prevalence in the U-shaped valley compared to the V-shaped valleys and at the plateau indicates that children in the U-shaped valley are regularly exposed to malaria parasites due to stable malaria transmission in these areas and, therefore, they have developed immunity to malaria parasites. Majority of residents in the U-shaped valleys suffer 2–6 episodes of malaria per year or are continuously infected for 6 months where as in the V-shaped valley and the plateau majority of the residents suffer 1–2 malaria episodes per year or are continuously infected for 2 months (42, 43). This shows that individual in the U-shaped valleys have developed immunity to malaria due to continuous exposure to malaria parasites. Similar studies have shown that the majority of breeding habitats in the hilly highlands are confined to the valley bottoms because the hillside gradients provide efficient drainage, this areas are, therefore, characterized by well-established transmission, with the majority of children 6–13 years old maintaining asymptomatic parasitemia (9, 10, 40). Previous studies using human sera from individuals in malaria endemic populations have found evidence of association between total IgG levels MSP2 with a reduced subsequent risk of clinical malaria (3, 19, 42–44). Other studies have shown that in regions where malaria is hyperendemic, adults develop potent but non-sterile immunity against malaria in which individuals chronically harbor low-grade parasitemia and only occasionally suffer from mild clinical malaria (53). Soe and others also showed that there is an association between antigen-specific antibody responses and protection against clinical malaria in Southeast Asia (53). Segeja and colleagues in Tanzania showed that individuals with higher levels of IgG are partially protected from malaria infections (54). The findings of our study indicate that the high prevalence of antibodies in the U-shaped valleys is responsible for asymptomatic malaria in these areas. In spite of high prevalence of malaria in the U-shaped valleys, individuals in the U-shaped valley have developed immunity to clinical malaria, individuals in the V-shaped valleys, on the other hand, have low prevalence of malaria but they also lack immunity to malaria and, therefore, they are more prone to malaria epidemics during hyper-transmission periods. Spatial analysis of the malaria antibodies and infections indicated that there was a significant positive clustering around swamps and marshes in the U-shaped valleys of western Kenya highlands.

## Conclusion

The findings of this study indicate that the topography characteristics of the highland valleys systems affect the exposure of the human population to malaria parasites and the immune response to malaria. The residents of V-shaped valleys are at risk of having explosive malaria outbreaks during hyper-transmission periods due to low exposure to malaria parasite; hence, they have low immune response to malaria, while the U-shaped valleys have stable malaria transmission; therefore, the human population has developed immunity to malaria due to continuous exposure to malaria. The spatial distribution maps have found clustering of malaria infections around the swamps and at low altitude in the U-shaped valleys and random distribution of malaria in the V-shaped valleys, implementation of malaria control treats the highlands as a homogeneous entity and does not take into consideration the high heterogeneity of transmission and incidence. Thus, the distribution of interventions does not take in care the different transmission intensities and disease incidence, topography maps can be reliably used to identify the affected areas and the scarce resources focused to these areas to control malaria.

## Author Contributions

CW conceived and designed the study, did data collection, and analysis. EK drafted the manuscript. Both revised and agreed upon submission.

## Conflict of Interest Statement

The authors declare that the research was conducted in the absence of any commercial or financial relationships that could be construed as a potential conflict of interest. The reviewer KS and handling editor declared their shared affiliation, and the handling editor states that the process nevertheless met the standards of a fair and objective review.

## Abbreviations

CSP, circumsporozoite protein; IgG, immuno globulin G; MSP, merozoite surface protein antibodies.

## References

[1] WHO. Factsheet on the World Malaria Report 2015. Geneva: World Health Organisation (2015).

[2] Malakooti MA Biomndo K Shanks GD. Reemergence of epidemic malaria in the highlands of western Kenya. Emerg Infect Dis (1998) 4(4):671–6. 10.3201/eid0404.980422 9866748 PMC2640260

[3] Shanks GD Biomndo K Hay SI Snow RW. Changing patterns of clinical malaria since 1965 among a tea estate population located in the Kenyan highlands. Trans R Soc Trop Med Hyg (2000) 94(3):253–5. 10.1016/S0035-9203(00)90310-9 10974991 PMC3272391

[4] Garnham P The incidence of malaria at high altitudes. J Natl Malar Soc (1948) 7(4):275–84. 18109262

[5] Roberts J The control of epidemic malaria in the highlands of western Kenya. Part II. The campaign. J Trop Med Hyg (1964) 67(8):191–9. 14179766

[6] Hay SI Cox J Rogers DJ Randolph SE Stern DI Shanks GD Climate change and the resurgence of malaria in the East African highlands. Nature (2002) 415(6874):905–9. 10.1038/415905a 11859368 PMC3164800

[7] Zhou G Minakawa N Githeko AK Yan G. Association between climate variability and malaria epidemics in the East African highlands. Proc Natl Acad Sci U S A (2004) 101(8):2375–80. 10.1073/pnas.0308714100 14983017 PMC356958

[8] Githeko AK Lindsay SW Confalonieri UE Patz JA. Climate change and vector-borne diseases: a regional analysis. Bull World Health Organ (2000) 78(9):1136–47. 11019462 PMC2560843

[9] Githeko AK Ayisi JM Odada PK Atieli FK Ndenga BA Githure JI Topography and malaria transmission heterogeneity in western Kenya highlands: prospects for focal vector control. Malar J (2006) 5(1):1–9. 10.1186/1475-2875-5-107 17096835 PMC1654174

[10] Atieli HE Zhou G Lee M-C Kweka EJ Afrane Y Mwanzo I Topography as a modifier of breeding habitats and concurrent vulnerability to malaria risk in the western Kenya highlands. Parasit Vectors (2011) 4(1):1–12. 10.1186/1756-3305-4-241 22196078 PMC3269397

[11] Rono J Färnert A Murungi L Ojal J Kamuyu G Guleid F Multiple clinical episodes of *Plasmodium falciparum* malaria in a low transmission intensity setting: exposure versus immunity. BMC Med (2015) 13(1):1–11. 10.1186/s12916-015-0354-z 25967134 PMC4445794

[12] Stevenson JC Stresman GH Baidjoe A Okoth A Oriango R Owaga C Use of different transmission metrics to describe malaria epidemiology in the highlands of western Kenya. Malar J (2015) 14(1):1–13. 10.1186/s12936-015-0944-4 26502920 PMC4624380

[13] Kangoye DT Mensah VA Murungi LM Nkumama I Nebie I Marsh K Dynamics and role of antibodies to *Plasmodium falciparum* merozoite antigens in children living in two settings with differing malaria transmission intensity. Vaccine (2016) 34(1):160–6. 10.1016/j.vaccine.2015.10.058 26541134 PMC4683095

[14] Himeidan YE-S Kweka E. Malaria in east African highlands during the past 30 years: impact of environmental changes. Front Physiol (2012) 3:315. 10.3389/fphys.2012.00315 22934065 PMC3429085

[15] Githeko AK Ototo EN Guiyun Y. Progress towards understanding the ecology and epidemiology of malaria in the western Kenya highlands: opportunities and challenges for control under climate change risk. Acta Trop (2012) 121(1):19–25. 10.1016/j.actatropica.2011.10.002 22015426 PMC3298846

[16] Wanjala CL Waitumbi J Zhou G Githeko AK. Identification of malaria transmission and epidemic hotspots in the western Kenya highlands: its application to malaria epidemic prediction. Parasit Vectors (2011) 4(1):1–13. 10.1186/1756-3305-4-81 21595898 PMC3117811

[17] Mouchet J Laventure S Blanchy S Fioramonti R Rakotonjanabelo A Rabarison P The reconquest of the Madagascar highlands by malaria. Bulletin de la Societe de pathologie exotique (1990) (1996) 90(3):162–8. 9410249

[18] Frosch AEP John CC. Immunomodulation in *Plasmodium falciparum* malaria: experiments in nature and their conflicting implications for potential therapeutic agents. Expert Rev Anti Infect Ther (2012) 10(11):1343–56. 10.1586/eri.12.118 23241191 PMC3632711

[19] Rolfes MA McCarra M Magak NG Ernst KC Dent AE Lindblade KA Development of clinical immunity to malaria in highland areas of low and unstable transmission. Am J Trop Med Hyg (2012) 87(5):806–12. 10.4269/ajtmh.2012.11-0530 22987652 PMC3516254

[20] John CC Ouma JH Sumba PO Hollingdale MR Kazura JW King CL. Lymphocyte proliferation and antibody responses to *Plasmodium falciparum* liver-stage antigen-1 in a highland area of Kenya with seasonal variation in malaria transmission. Am J Trop Med Hyg (2002) 66(4):372–8. 12164290 10.4269/ajtmh.2002.66.372

[21] Smith H Crandall I Prudhomme J Sherman I. Optimization and inhibition of the adherent ability of *Plasmodium falciparum*-infected erythrocytes. Memórias do Instituto Oswaldo Cruz (1992) 87:303–12. 10.1590/S0074-02761992000700052 1343707

[22] Snow R Craig M Newton C Steketee R The Public Health Burden of Plasmodium falciparum Malaria in Africa: Deriving the Numbers. The Disease Control Priorities Project (DCPP) Working Paper Number 11, 2003. Washington, DC (2008).

[23] Snow RW Omumbo JA Lowe B Molyneux CS Obiero J-O Palmer A Relation between severe malaria morbidity in children and level of *Plasmodium falciparum* transmission in Africa. Lancet (1997) 349(9066):1650–4. 10.1016/S0140-6736(97)02038-2 9186382

[24] Smith T Charlwood JD Kihonda J Mwankusye S Billingsley P Meuwissen J Absence of seasonal variation in malaria parasitaemia in an area of intense seasonal transmission. Acta Trop (1993) 54(1):55–72. 10.1016/0001-706X(93)90068-M 8103627

[25] Githeko AK Ndegwa W Predicting malaria epidemics in the Kenyan highlands using climate data: a tool for decision makers. Glob Change Hum Health (2001) 2(1):54–63. 10.1023/A:1011943131643

[26] Omumbo JA Lyon B Waweru SM Connor SJ Thomson MC. Raised temperatures over the Kericho tea estates: revisiting the climate in the East African highlands malaria debate. Malar J (2011) 10(1):1–16. 10.1186/1475-2875-10-12 21241505 PMC3031277

[27] Alonso D Bouma MJ Pascual M. Epidemic malaria and warmer temperatures in recent decades in an East African highland. Proc Biol Sci (2011) 278(1712):1661–9. 10.1098/rspb.2010.2020 21068045 PMC3081772

[28] Lindblade KA Walker ED Onapa AW Katungu J Wilson ML. Land use change alters malaria transmission parameters by modifying temperature in a highland area of Uganda. Trop Med Int Health (2000) 5(4):263–74. 10.1046/j.1365-3156.2000.00551.x 10810021

[29] Lindblade KA Walker ED Wilson ML. Early warning of malaria epidemics in African highlands using *Anopheles* (Diptera: Culicidae) indoor resting density. J Med Entomol (2000) 37(5):664–74. 10.1603/0022-2585-37.5.664 11004777

[30] Lindblade KA Walker ED Onapa AW Katungu J Wilson ML. Highland malaria in Uganda: prospective analysis of an epidemic associated with El Niño. Trans R Soc Trop Med Hyg (1999) 93(5):480–7. 10.1016/S0035-9203(99)90344-9 10696401

[31] Kweka EJ Zhou G Munga S Lee M-C Atieli HE Nyindo M Anopheline larval habitats seasonality and species distribution: a prerequisite for effective targeted larval habitats control programmes. PLoS One (2012) 7(12):e52084. 10.1371/journal.pone.0052084 23272215 PMC3525533

[32] Kweka EJ Kamau L Munga S Lee M-C Githeko AK Yan G. A first report of *Anopheles funestus* sibling species in western Kenya highlands. Acta Trop (2013) 128(1):158–61. 10.1016/j.actatropica.2013.06.006 23792011 PMC3775913

[33] Kweka EJ Zhou G Lee M-C Gilbreath TM Mosha F Munga S Evaluation of two methods of estimating larval habitat productivity in western Kenya highlands. Parasit Vectors (2011) 4(1):1–9. 10.1186/1756-3305-4-110 21682875 PMC3138440

[34] Minakawa N Sonye G Mogi M Yan G. Habitat characteristics of *Anopheles gambiae* s.s. larvae in a Kenyan highland. Med Vet Entomol (2004) 18(3):301–5. 10.1111/j.0269-283X.2004.00503.x 15347399

[35] Fillinger U Ndenga B Githeko A Lindsay SW. Integrated malaria vector control with microbial larvicides and insecticide-treated nets in western Kenya: a controlled trial. Bull World Health Organ (2009) 87:655–65. 10.2471/BLT.08.055632 19784445 PMC2739910

[36] Iqbal J Hira P Al-Ali F Khalid N Sher A. Modified Giemsa staining for rapid diagnosis of malaria infection. Med Princ Pract (2003) 12(3):156–9. 10.1159/000070751 12766332

[37] Slutsker L Taylor TE Wirima JJ Steketee RW. In-hospital morbidity and mortality due to malaria-associated severe anaemia in two areas of Malawi with different patterns of malaria infection. Trans R Soc Trop Med Hyg (1994) 88(5):548–51. 10.1016/0035-9203(94)90157-0 7992335

[38] Mitchell C Adebajo A Hay E Carr A Shoulder pain: diagnosis and management in primary care. BMJ (2005) 331(7525):1124–8. 10.1136/bmj.331.7525.1124 16282408 PMC1283277

[39] Himeidan YE Zhou G Yakob L Afrane Y Munga S Atieli H Habitat stability and occurrences of malaria vector larvae in western Kenya highlands. Malar J (2009) 8(1):1–6. 10.1186/1475-2875-8-234 19845968 PMC2771030

[40] Githeko AK Ogallo L Lemnge M Okia M Ototo EN. Development and validation of climate and ecosystem-based early malaria epidemic prediction models in East Africa. Malar J (2014) 13(1):1–11. 10.1186/1475-2875-13-329 25149479 PMC4158077

[41] Ototo EN Githeko AK Wanjala CL Scott TW Surveillance of vector populations and malaria transmission during the 2009/10 El Niño event in the western Kenya highlands: opportunities for early detection of malaria hyper-transmission. Parasit Vectors (2011) 4(1):1–9. 10.1186/1756-3305-4-144 21781291 PMC3148556

[42] Badu K Afrane YA Larbi J Stewart VA Waitumbi J Angov E Marked variation in MSP-119 antibody responses to malaria in western Kenyan highlands. BMC Infect Dis (2012) 12(1):1–9. 10.1186/1471-2334-12-50 22380785 PMC3306741

[43] Badu K Siangla J Larbi J Lawson BW Afrane Y Ong’echa J Variation in exposure to *Anopheles gambiae* salivary gland peptide (gSG6-P1) across different malaria transmission settings in the western Kenya highlands. Malar J (2012) 11(1):1–9. 10.1186/1475-2875-11-318 22963464 PMC3541077

[44] Afrane YA Zhou G Githeko AK Yan G. Clinical malaria case definition and malaria attributable fraction in the highlands of western Kenya. Malar J (2014) 13(1):1–7. 10.1186/1475-2875-13-405 25318705 PMC4209040

[45] Zhou G Munga S Minakawa N Githeko AK Yan G. Spatial relationship between adult malaria vector abundance and environmental factors in western Kenya highlands. Am J Trop Med Hyg (2007) 77(1):29–35. 17620627

[46] Stern DI Gething PW Kabaria CW Temperley WH Noor AM Okiro EA Temperature and malaria trends in highland East Africa. PLoS One (2011) 6(9):e24524. 10.1371/journal.pone.0024524 21935416 PMC3174181

[47] Baum E Badu K Molina DM Liang X Felgner PL Yan G. Protein microarray analysis of antibody responses to *Plasmodium falciparum* in Western Kenyan highland sites with differing transmission levels. PLoS One (2013) 8(12):e82246. 10.1371/journal.pone.0082246 24312649 PMC3846730

[48] Tchuinkam T Nyih-Kong B Fopa F Simard F Antonio-Nkondjio C Awono-Ambene H-P Distribution of *Plasmodium falciparum* gametocytes and malaria-attributable fraction of fever episodes along an altitudinal transect in Western Cameroon. Malar J (2015) 14(1):1–15. 10.1186/s12936-015-0594-6 25889511 PMC4354986

[49] Gupta S Snow RW Donnelly CA Marsh K Newbold C. Immunity to non-cerebral severe malaria is acquired after one or two infections. Nat Med (1999) 5(3):340–3. 10.1038/6560 10086393

[50] Bødker R Msangeni HA Kisinza W Lindsay SW. Relationship between the intensity of exposure to malaria parasites and infection in the Usambara Mountains, Tanzania. Am J Trop Med Hyg (2006) 74(5):716–23. 16687668

[51] Bousema JT Drakeley CJ Mens PF Arens T Houben R Omar SA Increased *Plasmodium falciparum* gametocyte production in mixed infections with *P. malariae*. Am J Trop Med Hyg (2008) 78(3):442–8. 18337341

[52] Protopopoff N Van Bortel W Marcotty T Van Herp M Maes P Baza D Spatial targeted vector control is able to reduce malaria prevalence in the highlands of Burundi. Am J Trop Med Hyg (2008) 79(1):12–8. 18606758

[53] Soe S Theisen M Roussilhon C Aye K-S Druilhe P. Association between protection against clinical malaria and antibodies to merozoite surface antigens in an area of hyperendemicity in Myanmar: complementarity between responses to merozoite surface protein 3 and the 220-kilodalton glutamate-rich protein. Infect Immun (2004) 72(1):247–52. 10.1128/iai.72.1.247-252.2004 14688102 PMC343946

[54] Segeja MD Mmbando BP Seth MD Lusingu JP Lemnge MM. Acquisition of antibodies to merozoite surface protein 3 among residents of Korogwe, north eastern Tanzania. BMC Infect Dis (2010) 10(1):1–7. 10.1186/1471-2334-10-55 20205959 PMC2841183

